# SARS-CoV-2 atypical viral pneumonia with 25/25 computed tomography score

**DOI:** 10.11604/pamj.2021.39.161.30177

**Published:** 2021-07-01

**Authors:** Moli Jai Jain, Vishnu Vardhan

**Affiliations:** 1 Department of Cardiovascular and Respiratory Physiotherapy Sciences, Ravi Nair Physiotherapy College, Datta Meghe Institute of Medical Sciences, Wardha, Maharashtra, India

**Keywords:** Atypical viral pneumonia, SARS-CoV-2, COVID-19, high-resolution computed tomography

## Image in medicine

A-36-year-old female patient came to the emergency department with the complaints of fever with chills, along with progressing breathing difficulty since 8 days. She was advised rapid antigen test on 22/04/2021 and a sample was taken for reverse transcription polymerase chain reaction (RT-PCR), both came out to be positive for SARS-CoV-2. Following this, her high-resolution computed tomography (HRCT) thorax (A) was done which reveals multiple ill-defined patchy ground-glass opacities with consolidation, septal thickening, and fibrotic changes in bilateral lung fields as described above signs suggestive of COVID-19 changes with atypical viral pneumonia. The computed tomography (CT) severity score was 25/25 (severe) with COVID-19 Reporting and Data System (CO-RADS) grade 6. The patient was immediately admitted to the isolation intensive care unit (ICU). High flow O_2_ support was started as SpO_2_ on admission was 44% on room air. Despite high flow of O_2_, patient was breathless and SpO_2_ was 57%. She was hence taken on bilevel positive airway pressure (BiPAP) with a 14-8 setting with O_2_ support. Along with medical management, physiotherapy treatment was administered. After 22 days the RT-PCR was reported negative and was shifted to high dependency unit and advised chest X-ray (B) which suggestive of bilateral opacities seen in bilateral lobes and right hemi diaphragm is pulled up. She received regular physiotherapy and was gradually wean off from ventilator and was maintaining saturation at the flow rate of 15 litre O_2_/min. Early administration of rehabilitation program will help in prevent further complications among such patients.

**Figure 1:**
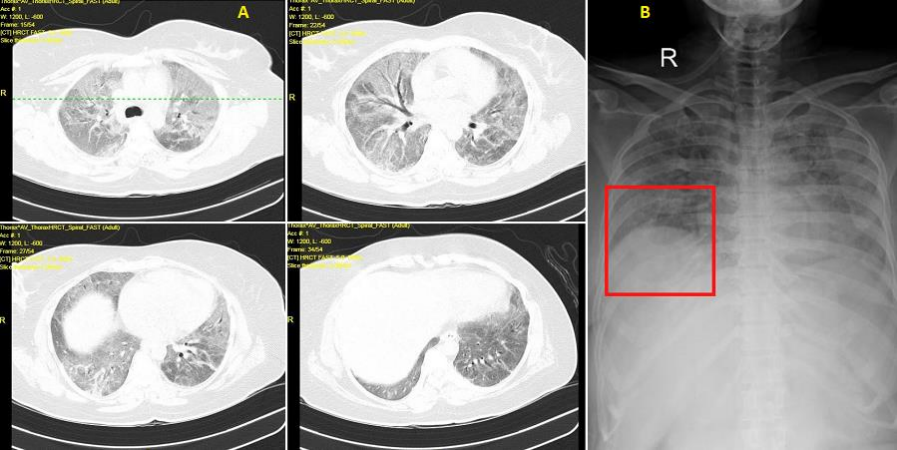
A) multiple ill-defined patchy ground-glass opacities with consolidation, septal thickening, and fibrotic changes in bilateral lung fields suggestive of COVID-19 changes with atypical viral pneumonia with CT severity score 25/25; B) bilateral opacities seen in bilateral lobes and right hemi diaphragm is pulled up

